# Comparison of MR‐soft tissue based versus biliary stent based alignment for image guidance in pancreatic SBRT

**DOI:** 10.1002/acm2.13965

**Published:** 2023-03-16

**Authors:** Zhaohui Han, Atchar Sudhyadhom, Shu‐Hui Hsu, Yue‐Houng Hu, Raymond H. Mak, Mai Anh Huynh, Ritchell R. van Dams, Shyam Tanguturi, Veena Venkatachalam, Joseph D. Mancias, Harvey J. Mamon, Neil E. Martin, Miranda B. Lam, Jonathan E. Leeman

**Affiliations:** ^1^ Department of Radiation Oncology Brigham and Women's Hospital and Dana‐Farber Cancer Institute Harvard Medical School Boston Massachusetts USA

**Keywords:** biliary stent, image guidance, MR‐guided radiation therapy, pancreatic SBRT

## Abstract

**Purpose:**

The role of biliary stents in image‐guided localization for pancreatic cancer has been inconclusive. To date, stent accuracy has been largely evaluated against implanted fiducials on cone beam computed tomography. We aim to use magnetic resonance (MR) soft tissue as a direct reference to examine the geometric and dosimetric impacts of stent‐based localization on the newly available MR linear accelerator.

**Methods:**

Thirty pancreatic cancer patients (132 fractions) treated on our MR linear accelerator were identified to have a biliary stent. In our standard adaptive workflow, patients were set up to the target using soft tissue for image registration and structures were re‐contoured on daily MR images. The original plan was then projected on treatment anatomy and dose predicted, followed by plan re‐optimization and treatment delivery. These online predicted plans were soft tissue‐based and served as reference plans. Retrospective image registration to the stent was performed offline to simulate stent‐based localization and the magnitude of shifts was taken as the geometric accuracy of stent localization. New predicted plans were generated based on stent‐alignment for dosimetric comparison.

**Results:**

Shifts were within 3 mm for 90% of the cases (mean = 1.5 mm); however, larger shifts up to 7.2 mm were observed. Average PTV coverage dropped by 1.1% with a maximum drop of 26.8%. The mean increase in V35Gy was 0.15, 0.05, 0.02, and 0.02 cc for duodenum, stomach, small bowel and large bowel, respectively. Stent alignment was significantly worse for all metrics except for small bowel (*p* = 0.07).

**Conclusions:**

Overall discrepancy between stent‐ and soft tissue‐alignment was modest; however, large discrepancies were observed for select cases. While PTV coverage loss may be compensated for by using a larger margin, the increase in dose to gastrointestinal organs at risk may limit the role of biliary stents in image‐guided localization.

## 1 INTRODUCTION

Pancreatic cancer ranks fourth among cancer deaths in the United States.^1^ At presentation, less than 20% of pancreatic cancer patients are surgical candidates for a chance of potential cure.^2^ For patients with unresectable or borderline resectable tumors, the current treatment option includes chemotherapy, often followed by radiation therapy.^3^ The major challenges of delivering radiation therapy, particularly using high dose stereotactic body radiotherapy (SBRT), are toxicities to the adjacent organs at risk (OAR) including stomach, duodenum and other parts of the small bowel.^4^, ^5^ High confidence in image guidance and localization is therefore crucial in the management of pancreatic cancer using radiation.

Traditionally, radiation treatments of pancreatic cancer are delivered on a conventional linear accelerator (linac) using cone beam computed tomography (CBCT) for image‐guided localization. Due to the lack of soft issue resolution on CBCT images, implanted fiducials are typically required for image registration.^6^ In the absence of fiducials, biliary stents are often used as a surrogate.^7^ The use of biliary stents for localization has been controversial but nonetheless remains a common clinical practice, especially when there is no institutional access to fiducial markers.^7^ Different degrees of geometric discrepancy have been reported for stent versus fiducials based on CBCT analysis.^8^, ^9^, ^10^ However, agreement or discrepancy for stent versus soft tissue, which ideally should serve as the true reference, has yet to be reported in the literature, largely because of the limited soft tissue detectability on CBCT.

The newly available magnetic resonance (MR) imaging capability on a linac allows for soft tissue‐based contouring, image guidance and online adaptation.^11^, ^12^ While fiducial or stent placement is not necessary for treatment on MR linac systems, some pancreatic cancer patients treated on our MR linac already have a biliary stent placed prior to referral for SBRT. Regardless of the presence of a stent, patients are set up using soft tissues on MR for alignment, which could provide a soft issue‐based reference. In this work, we aim to take advantage of the availability of both soft tissue and stents for image registration on our MR linac platform to evaluate the discrepancy between stent versus soft tissue alignment for pancreatic SBRT. Additionally in a standard online adaptive workflow, target and OARs are recontoured by a radiation oncologist on daily MR images to account for inter‐fractional anatomy changes.^13^, ^14^ Recontouring allows for dosimetric evaluation of different image alignments on the same structure set using treatment day anatomy, which has not been previously possible with CBCT based modalities.

## 2 METHODS AND MATERIALS

### 2.1 Patients and treatment protocols

In this retrospective study approved by the institutional review board, 30 pancreatic cancer patients treated between 2019 and 2022 on our ViewRay MRIdian MR linac (ViewRay Inc., Oakwood Village, OH) were identified to have a biliary stent. All patients had pancreatic head tumors and the stent was near or partially inside the treatment volume. The necessity of the stent was for the relief of biliary duct obstruction only and was not placed for radiation treatment purposes.

All patients were treated with SBRT to a total of 40 Gy or 33 Gy over five fractions. Two weeks prior to initiating radiation treatment, patients underwent both MR and computed tomography (CT) simulations under breath hold and an initial radiation plan was developed by a medical dosimetrist. The MR scan was acquired on our MR linac with a magnetic field strength of 0.35 T using a TrueFISP sequence.^15^ The MR images were primarily used for target/OAR delineation and as the reference images for daily patient setup. The CT images were primarily used to provide electron density for dose calculations and could be used to assist contouring. A 3 mm isotropic margin was used for planning target volume (PTV) expansion. The primary PTV, excluding gastrointestinal (GI) organs, was typically prescribed to be covered to ≥90% at the prescription dose. GI OAR metrics included V35Gy < 0.03cc and V33Gy < 0.5cc which were considered “hard constraints” and had to be met. In our treatment protocol, compromise to PTV coverage was allowed in order to meet the more stringent GI OAR metrics. All treatments were planned with IMRT (intensity modulated radiation therapy) technique, typically with 15–20 beams. Dose calculation was done through a Monte Carlo algorithm implemented in the ViewRay planning system which accounted for the presence of the magnetic field. The dose grid was 2 mm and calculation uncertainty was 0.5%.

**FIGURE 1:**
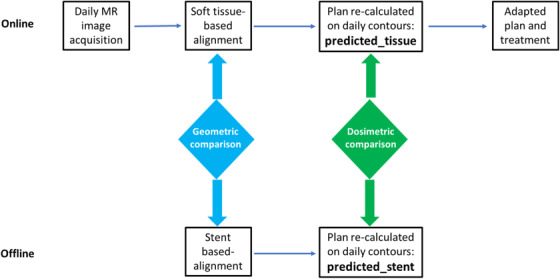
Methodologies of this study: Top row, online adaptive workflow which provides a soft tissue‐based reference; Bottom row, offline retrospective study using biliary sent for alignment which simulates the standard practice of managing pancreatic cancer with a biliary stent in a non‐adaptive sitting, for example, on a regular linac where the majority of the pancreatic cases are treated. The comparison of stent‐alignment against soft tissue‐aligned reference plans was evaluated both geometrically and dosimetrically.

The methodologies of this study are illustrated in Figure 1. In the top row of Figure 1 is our online adaptive workflow which provides a soft tissue‐based reference; In the bottom row of Figure 1 is an offline study using sent for alignment, which simulates the standard practice of managing pancreatic cancer with a biliary stent on a regular linac where the majority of the pancreatic cases are treated. The comparison between stent‐ versus soft tissue‐based alignments was evaluated both geometrically and dosimetrically.

### 2.2 Online soft tissue‐based alignment

All patients were managed with an online adaptive workflow as shown in the top row of Figure 1. Treatment day MR was acquired after the patient had been positioned on the treatment table as determined at simulation. The initial planning MR was then aligned to the daily MR, focusing on the target using soft tissue contrast. After patients were shifted to the treatment position, original contours and electron density were deformed to daily MR images. Additional adjustments to contours based on daily anatomy were done by a radiation oncologist as needed, followed by adjustments to electron density by a medical physicist. The original plan was then projected on the daily anatomy and dose was re‐calculated. These re‐calculated plans are referred to as “predicted” plans on ViewRay system and hereinafter, we refer the online predicted plans as “predicted_tissue” since the image registration was based on soft tissue alignment. These “predicted_tissue” plans will serve as the reference in this study. Although predicted plans were typically not delivered on our adaptive MR linac platform, they would have represented the delivery of the original plans to patients in a non‐adaptive setting (e.g., on a regular linac), with added benefits that the imaging localization was soft tissue‐based and the delivered dose could be evaluated on treatment anatomy thanks to daily recontouring.

In our clinical adaptive workflow, treatment plans were further optimized and adapted to daily anatomy before they were delivered as shown in the final step in the top row of Figure 1. Plan adaptation is beyond the scope of this presentation and therefore will not be studied in detail; however, some statistics of the adapted plans is included in this study to provide additional information.

### 2.3 Offline stent‐based alignment and geometric evaluations

The offline workflow was illustrated in the bottom row of Figure 1 which started with retrospective registration of planning to daily MR images using biliary stents for alignment. Stent registration was performed for the 30 patients who had a stent for a total of 132 fractions. The use of a biliary stent for image alignment simulates the common practice of managing pancreatic cancer with a biliary stent on a regular linac. The registration was done in the ViewRay treatment planning system by a medical physicist and reviewed by a radiation oncologist. The registration mimicked typical clinical practice on a regular linac for pancreas SBRT, which included translational shifts only with a focus on regions near the treatment site. The required additional shifts to align the stent (from the already aligned soft tissue online) represented the geometric discrepancy of stent‐based registration compared to soft tissue registration. To facilitate a comparison with published data, the magnitude of the three‐dimensional (3D) shifts was calculated and reported in this study.

### 2.4 Dosimetric analysis

The offline workflow also generated a second predicted plan based on stent alignment (hereinafter “predicted_stent”) for each of the 132 fractions as shown in the bottom row of Figure 1. The plan metrics based on stent alignment were evaluated against the online soft tissue aligned reference plans. Compared with predicted_tissue, predicted_stent plans had an isocenter shift which was a direct result of the geometric discrepancy of stent versus soft tissue alignment. Both predicted plans were calculated on the same anatomy and structure set with identical planning parameters including total monitor units, beam geometry, dose grid of 2 mm, and calculation uncertainties of 0.5% with magnetic field accounted for. Therefore, any dosimetric discrepancies between predicted_stent and predicted_tissue plans were directly attributable to the different alignment methods.

Dosimetric evaluation included target coverage and OAR sparing. In this work, we focused on the primary PTV coverage at prescription dose (V100%) and GI OAR volumes receiving ≥35 Gy (V35Gy), which was capped at 0.03 cc for duodenum, stomach, small bowel and large bowel in our treatment protocols. All analysis was carried out in ViewRay planning system. The significance of the dosimetric changes from soft tissue alignment to stent alignment was determined using a paired t‐test, where *p* < 0.05 was considered statistically significant.

## 3 RESULTS

### 3.1 Stent registration and geometric results

**FIGURE 2:**
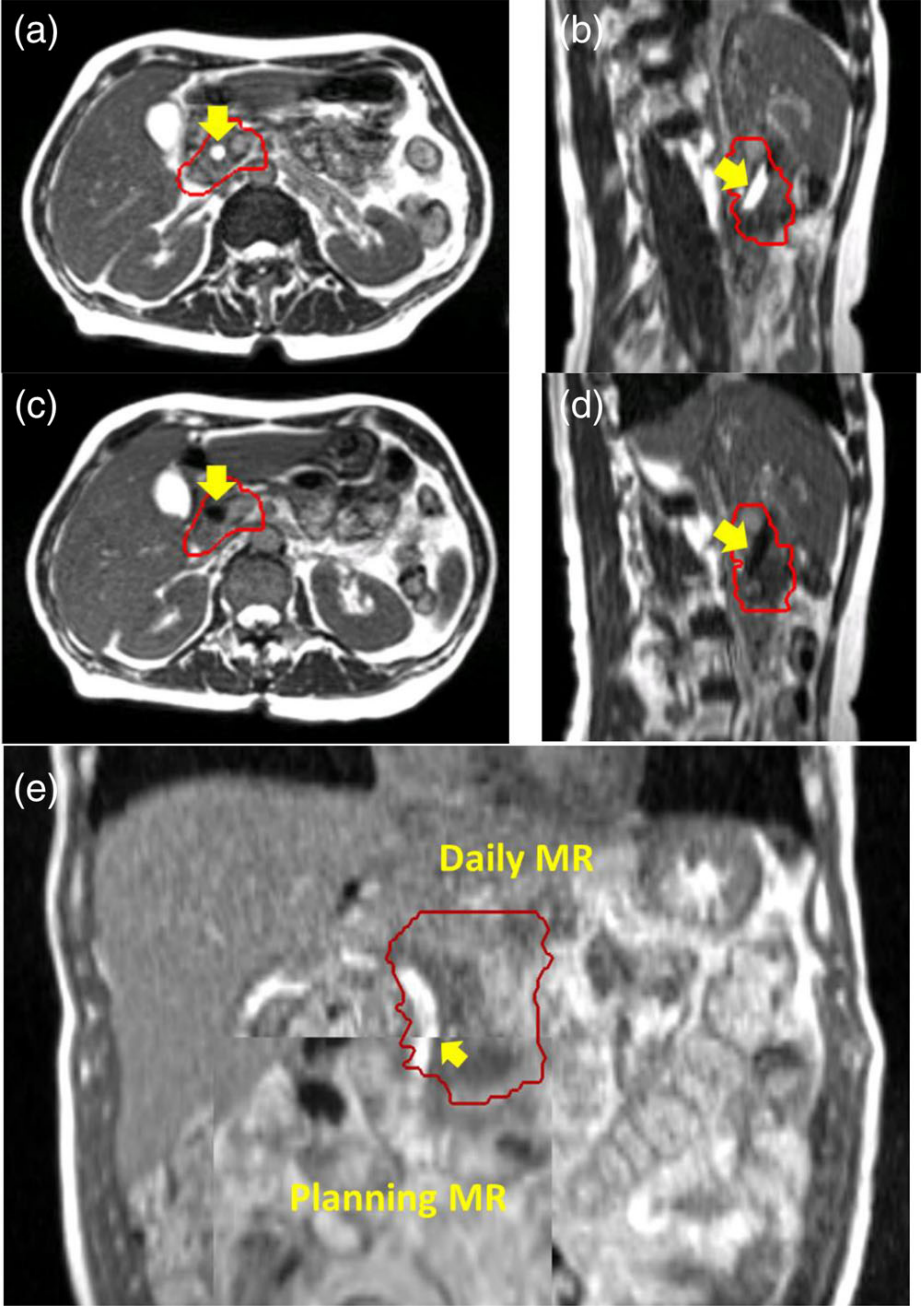
The appearance of the biliary stent (arrows) varied on TrueFISP MR images depending on daily stent filling. It appeared bright when filled with liquid (A and B) and dark when filled with air (C and D). When registered with soft tissue online, the stent can be misaligned (arrow in E). Red contours show the PTV.

The appearance of a biliary stent varied on a TrueFISP MR image depending on daily stent filling. Figure 2A–D shows an example for two consecutive treatment fractions for the same patient. The stent appeared bright when filled with liquid (A, axial view; B, sagittal view), and dark when filled with air (C, axial view; D, sagittal view). Figure 2E is a split‐window view of a registration between planning and daily MR images. In this coronal view, the stent was clearly misaligned when online image registration was based on soft tissue alignment.

**FIGURE 3:**
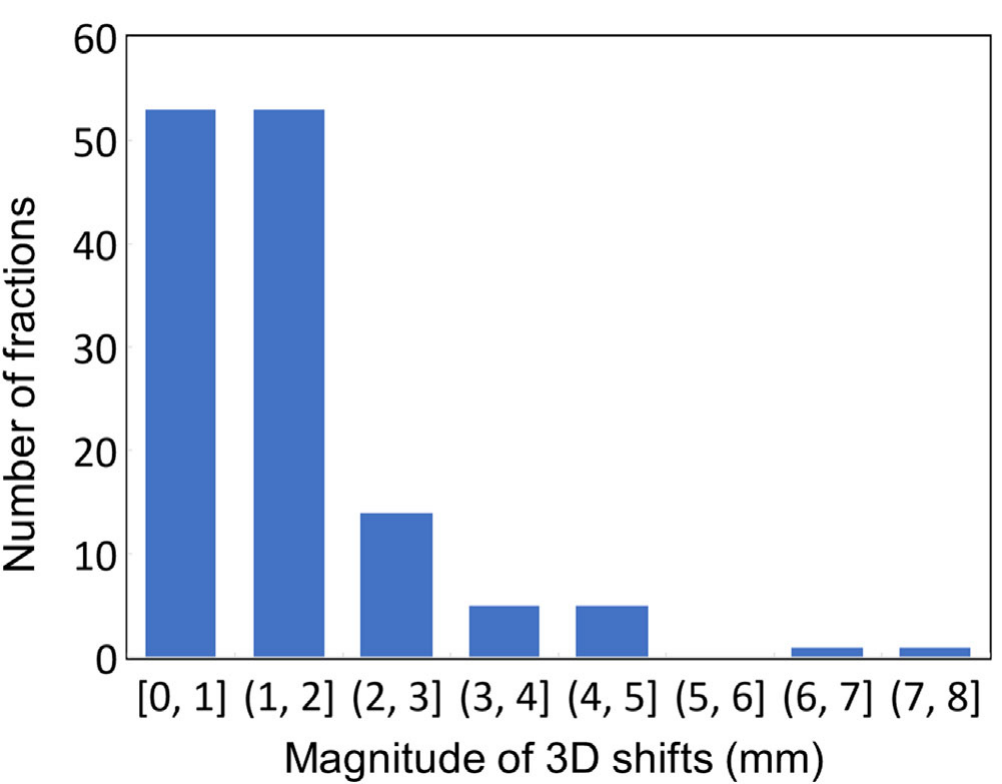
Magnitude of 3D shifts from soft tissue alignment to stent alignment. The stent registration was focused on regions near the treatment site mimicking our clinical practice on a CBCT‐based linac. The mean shift was 1.5 mm and the range was 0–7.2 mm. In 10% of the fractions, greater than 3 mm shifts were observed.

Figure 3 is the histogram of the magnitude of 3D shifts from soft tissue alignment to stent alignment, representing the geometric discrepancy between these two methods of localization. While the discrepancy was less than 3 mm in the majority of fractions (90%), a discrepancy with a magnitude as large as 7.2 mm was observed. The mean discrepancy was 1.5 mm (range 0–7.2 mm).

### 3.2 Dosimetric results

**FIGURE 4:**
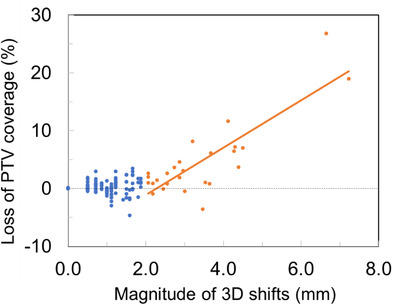
Loss in PTV coverage as a function of 3D shifts. A positive correlation existed for shifts greater than 2 mm (orange). No correlation was observed for shifts less than 2 mm (blue), possibly obscured by the dose calculation grid of 2 mm used.

**FIGURE 5:**
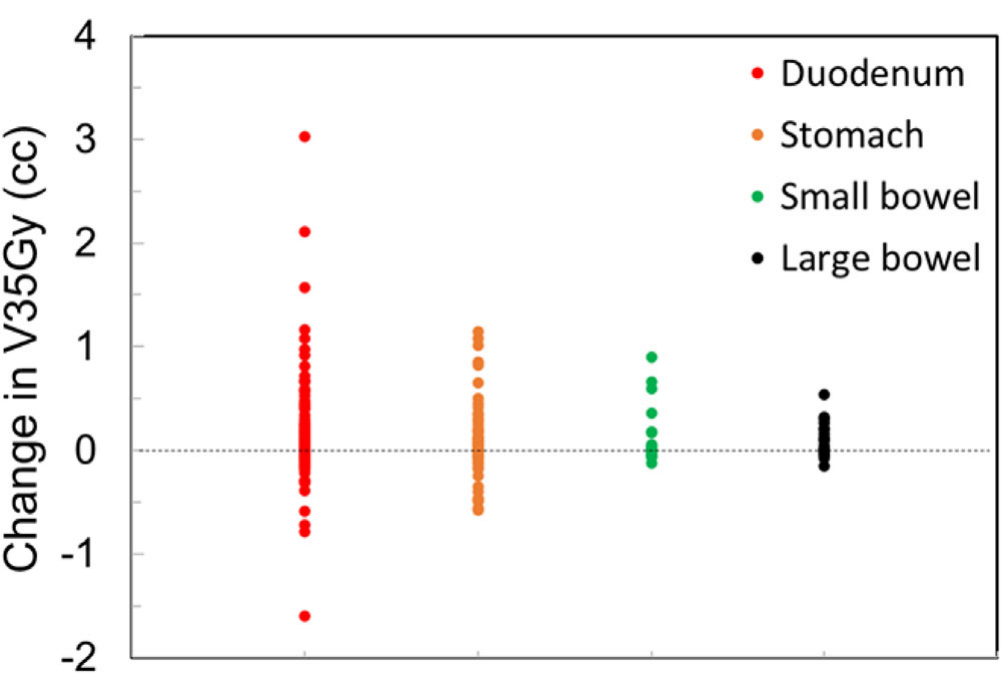
Increase in V35Gy for GI OARs from soft tissue‐ to stent‐based localization. The average increase was 0.15, 0.05, 0.02 and 0.02 cc for duodenum, stomach, small bowel and large bowel, respectively. Stent localization was significantly worse for all organs except for small bowel (*p* = 0.07).

Table 1 summarizes the mean and difference (Δ = predicted_stent—predicted_tissue) for a variety of dosimetric quantities. While the average loss in PTV coverage for all 132 fractions was small at 1.1%, the largest observed loss of PTV coverage was 26.8%. Figure 4 shows the loss in primary PTV coverage as a function of the magnitude of 3D shifts. A positive correlation existed for shifts greater than 2 mm. A linear fit (*R* ^2^ = 0.7) gave a loss rate of about 4% per mm as shown as a solid line in Figure 4. No correlation was observed for shifts less than 2 mm. As also presented in Table 1, the average increase in V35Gy from soft tissue‐ to stent‐alignment was 0.15, 0.05, 0.02, and 0.02 cc for duodenum, stomach, small bowel and large bowel, respectively. The distribution of the increases is shown in Figure 5. Paired *t*‐test showed that stent alignment was significantly worse for all metrics except for small bowel V35Gy (*p* = 0.07), indicating that localization using soft tissue better matched daily anatomy. However, there were some negative data points in Figures 4 and 5, indicating that stent alignment was better in those cases.

**TABLE 1:** Dosimetric comparison of soft tissue‐ and stent‐alignment.

Metric	Mean			Δ		
	Tissue	Stent	Adapted	mean	min	max
PTV V100% (%)	86.65%	85.55%	90.9%	−1.1%	4.66%	−26.8%
PTV Dmin (Gy)	25.17	24.67	28.34	−0.5	4.43	−5.86
Duodenum V35Gy (cc)	0.95	1.10	0.01	0.15	−1.6	3.03
Stomach V35Gy (cc)	0.41	0.47	0.006	0.05	−0.58	1.14
Small Bowel V35Gy (cc)	0.05	0.07	0.003	0.02	−0.12	0.9
Large Bowel V35Gy (cc)	0.13	0.15	0.002	0.02	−0.15	0.54

PTV V100%: Percentage PTV covered at the prescription dose.

PTV Dmin: Minimum dose in PTV.

V35Gy: Organ volumes receiving ≥35 Gy.

Tissue: Predicted plan based on soft tissue alignment, predicted_tissue.

Stent: Predicted plan based on stent alignment, predicted_stent.

Adapted: Plan adapted to treatment anatomy online.

Δ: Metric difference between stent‐ and soft tissue‐alignment (Δ = predicted_stent—predicted_tissue).

Table 1 also shows the mean PTV and OAR metrics for the adapted plans to provide additional information. Compared with predicted_tissue plans, online adaptation was able to meet GI OAR constraints in all 132 fractions while improving PTV coverage in 103 fractions (78.0%). The mean improvement was 4.3%.

## 4 DISCUSSION

The adequacy of using biliary stents to characterize pancreatic tumor motion/position has been inconclusive in the literature. While some studies called for caution,^16^ others found the biliary stent a reasonable surrogate for pancreatic tumor, particularly when placed close to tumor.^17^ In the image‐guided radiotherapy setting, the use of biliary stents for tumor targeting and localization has also been subject to debate but nonetheless remains a common clinical practice in the absence of implanted fiducials.^7^, ^8^, ^9^, ^10^ SBRT has been playing an increasing role in the management of pancreatic cancer due to its demonstrated benefits.^18^ Dose escalation in SBRT requires high confidence in tumor localization due to the adjacent radiosensitive GI OARs, which often abut tumor. In this context, the accuracy of using a stent as a surrogate for pancreatic tumor localization was previously studied with reported accuracy ranging from 2 mm to >10 mm.^8^, ^9^, ^10^ All of the previous studies were based on CBCT guidance as pancreatic SBRT has been traditionally treated on conventional linacs. Due to the lack of soft tissue contrast on CBCT, fiducial markers were typically implanted and were taken as the “gold standard” to evaluate stent accuracy. The recent arrival of MR linacs in clinics allows for soft tissue‐based localization in pancreatic SBRT.^19^ MR also allows for examination of stent accuracy using soft tissue as a “true” reference in a subgroup of patients who had a biliary stent placed for relief of bile duct obstruction. To the best of our knowledge, there have been no previous reports of using images from an MR Linac to evaluate the use of biliary stents as a surrogate marker. In addition, pancreatic cancer patients are typically treated with an online adaptive workflow on our ViewRay platform and contours are re‐drawn on localization MR images, allowing for dosimetric study of stent‐versus soft tissue‐alignment on treatment anatomy which has not been possible in the past.

All pancreatic cancer patients who had a biliary stent and were treated on our MR linac had tumors located in pancreatic head. Typically, the stent was partially inside or abutting the PTV as shown in Figure 2. The different appearance of stent due to different filling (air versus liquid) can lead to some registration uncertainties, which can be mitigated by proper use of “gray” and “inverse gray” modes that are available in the ViewRay planning system. In retrospective stent alignment, we focused on the portion of stent that was close to the PTV to simulate the actual clinical practice. For the majority of our cases (90%), geometric discrepancy between stent and soft tissue was within 3 mm, the typical PTV margin used for pancreatic cases on our MR linac; however, a larger discrepancy was observed for about 10% of our cases. The image resolution difference between our daily MR (1.5 × 1.5 × 3 mm) and a typical abdomen CBCT (1 × 1 × 2 mm) on our Varian linacs may introduce some uncertainty in the geometric analysis. This uncertainty is estimated to be on the order of 1 mm. Overall, inaccuracy with stent alignment observed in our study seemed to be on the lower side of a wide spectrum of reported values,^8^, ^9^, ^10^ which may be attributable to the fact that all of our pancreatic cases were managed with breath hold on our MR linac where respiratory motion was minimized. In contrast, larger values reported earlier were all based on free breathing CBCT.^8^, ^9^ Stent motion due to respiration can be substantial on the order 10 mm in 3D.^20^ In addition, differential motion was also reported for stent and target,^20^ possibly further complicating the analysis on free breathing CBCT. If, as we hypothesize, respiratory motion accounts for a substantial portion of the variability in stent position, pancreatic tumors would be more accurately localized with CBCT acquired under breath hold or respiratory gating when a stent is used for alignment.

Other factors may also contribute to the smaller stent inaccuracy observed in our study. The earlier CBCT studies used the entire stent from which a centroid point was derived to represent the stent location.^8^, ^9^ In our clinical practice where stent was used for localization, manual registration was typically used with a focus on areas near the target. The same procedure was followed in the current study to reflect the actual clinical practice in our institute. We have noticed that in some cases, the stent deformed and showed daily variations, which would make registration to the entire stent difficult and potentially result in different shifts than focusing on regions near the target. Lastly, our study directly compared stent with soft tissue, whereas CBCT studies used implanted fiducials as an intermediate reference.^8^, ^9^ In addition, implanted fiducials can be subject to migration and respiratory motion on free breathing CBCT as well.^9^

While the overall loss in PTV coverage was modest (mean = 1.1%) when re‐aligned to stent from soft tissue, large drop up to 26.8% was observed in a fraction of cases as shown in Figure 4, indicating that breath hold alone may not account for the discrepancy between soft tissue versus stent alignments. For shifts > 2 mm, the loss showed positive correlation with geometric shifts which was interestingly absent for shifts < 2 mm. We suspect that the seemingly lack of a correlation for shifts < 2 mm was obscured by the dose calculation grid of 2 mm coupled with data processing and analysis noise, as well as the uncertainty threshold used in the Monte Carlo dose calculation. The choice of 2 mm grid size was a balanced consideration between dosimetric accuracy and adaptive efficiency. A further decrease in dose grid, which may modestly improve dosimetric accuracy, will result in a considerable increase in calculation time and therefore patient time on the treatment couch.

Unlike the overall small drop in PTV coverage, the increase in GI OAR dose was substantial, especially for duodenum and stomach where V35Gy increased by 0.15 cc and 0.05 cc on average, respectively. While the loss of tumor coverage may be compensated for using a larger PTV margin, it might be more difficult to manage the increased OAR dose non‐adaptively. As evident in Table 1, online adaptation successfully met V35Gy < 0.03cc for all GI OARs and may represent the best option for pancreatic cancers if available.

The limitations of our study include the lack of six degree‐of‐freedom (DoF) corrections in image registrations on the MR linac platform. Six DoF capability has become more widely available on regular linacs in the past few years and has been gradually adopted in the management of pancreatic SBRT in many clinics. While our findings can provide direct information in the context of three DoF corrections, further studies to quantify the differences between three DoF and six DoF on regular linacs are highly desirable to gain a full understanding of stent accuracy under six DoF corrections. In addition, there are variations among clinics with regard to using the stent for localization. Some clinics use the entire stent while others use the portion of stent near the target, with or without including surrounding soft tissue. It is beyond the scope of this study to address these variations.

## 5 CONCLUSIONS

The geometric and dosimetric impacts of using biliary stents for image guided localization in pancreatic SBRT were evaluated using soft tissue and treatment day anatomy as a reference. The overall geometric discrepancy between stent and soft tissue was found to be small, which may be attributable to the fact that all cases in our study were treated with breath hold. Caution must be exercised, though, as a larger discrepancy was observed for a fraction of cases. When re‐aligned to stent from soft tissue, the overall loss in PTV coverage was modest and may be compensated for by using a larger PTV margin; however, the increase in GI OAR dose was significant and may limit the role of biliary stent in image‐guided localization for pancreatic cancers in a non‐adaptive setting.

## AUTHOR CONTRIBUTIONS

All listed authors contributed to the study and to drafting and revising the manuscript.

## CONFLICT OF INTEREST STATEMENT

Zhaohui Han PhD receives research support and consulting fees from ViewRay Inc.

Raymond H. Mak MD receives research grants, consulting fees, and travel expenses from ViewRay Inc., consulting fees from AstraZeneca, speaker honoraria from Novartis, AAPM and King Faisal Specialist Hospital and paid expert testimony from US Attorneys for the District of New York; is co‐founder of HealthAI and serves on the editorial board of Communications Medicine.

Mai Anh Huynh, MD PhD receives research support and consulting fees from ViewRay Inc., Harvey J.

Mamon MD PhD receives royalties from UpToDate and consulting fees from Merck.

Jonathan E. Leeman MD receives research grants from ViewRay Inc. and NH TherAguix.

## Data Availability

Data supporting the findings of this study are stored on an institutional shared drive and are available upon request from the corresponding author with appropriate anonymization of patient information.
